# Ambiguity-aware semi-supervised learning for leaf disease classification

**DOI:** 10.1038/s41598-025-95849-3

**Published:** 2025-04-23

**Authors:** Tri-Cong Pham, Tien-Nam Nguyen, Van-Duy Nguyen

**Affiliations:** 1 Thuyloi University, 175 Tay Son, Dong Da, Hanoi, 10000 Vietnam; 2 L3i Laboratory, University of La Rochelle, 17000 La Rochelle, France; 3 Institute of Biotechnology and Environment, Nha Trang University, Khanh Hoa, Vietnam; 4 School of Engineering, Newcastle University, Newcastle upon Tyne, UK

**Keywords:** Deep learning, Semi-supervised learning, Ambiguity rejection, Leaf disease classification, Coffee leaf disease, Banana leaf disease, Engineering, Biomedical engineering, Bioinformatics

## Abstract

In deep learning, Semi-Supervised Learning is a highly effective technique to enhances neural network training by leveraging both labeled and unlabeled data. This process involves using a trained model to generate pseudo labels to the unlabeled samples, which are then incorporated to further train the original model, resulting in a new model. However, if these pseudo labels contain substantial errors, the resulting model’s accuracy may drop, potentially falling below the performance of the initial model. To tackle the problem, we propose an Ambiguity-Aware Semi-Supervised Learning method for Leaf Disease Classification. Specifically, we present a per-disease ambiguity rejection algorithm that eliminates ambiguous results, thereby enhancing the precision of pseudo labels for the subsequent semi-supervised training step and improving the precision of the final classifier. The proposed method is evaluated on two public leaf disease datasets of coffee and banana across various data scenarios, including supervised and semi-supervised settings, with varying proportions of labeled data. The results indicate that our semi-supervised method reduces the reliance for fully labeled datasets while preserving high accuracy by utilizing the ambiguity rejection algorithm. Additionally, the rejection algorithm significantly boosts precision of final classifier on both coffee and banana datasets, achieving rates of 99.46% and 100.0%, respectively, while using only 50% labeled data. The study also presents a thorough set of experiments and analyses to validate the effectiveness of the proposed method, comparing its performance against state-of-the-art supervised approaches. The results demonstrate that our method, despite using only 50% of the labeled data, achieves competitive performance compared to fully supervised models that use 100% of the labeled data.

## Introduction

Agriculture plays a pivotal role in the global economy, extending beyond the cultivation of rice, wheat or corn to encompass a wide range of crops essential for human sustenance and economic stability. As the backbone of many developing nations, agriculture not only provides food, but also serves as a primary source of income and employment for millions. Countries like Brazil, Indonesia, India, VietNam, Thailand, United States, and Russian have long been leaders in the production of key agricultural commodities such as coffee, rice, corn, wheat which are one the most traded goods worldwide. Such advancements in agriculture contribute significantly to a nation’s economic growth, enhancing both the quality and quantity of agricultural output. Despite these advancements, the agricultural sector continues to face numerous challenges, including the impact of climate change, the emergence of plant diseases. These issues underscore the importance of continuous innovation and investment in agricultural technologies to ensure sustainable production and to safeguard the livelihoods of those dependent on this crucial industry. Sustainable practices include the adoption of eco-friendly farming techniques, such as crop diversification, organic farming. These methods not only protect the soil and biodiversity but also contribute to the resilience of plants against pests and diseases. The world’s rapidly changing environment is contributing to the emergence of numerous leaf diseases, adversely affecting both the quality and quantity of agricultural production and ultimately reducing revenue. A major challenge in leaf disease diagnosis is the agricultural sector’s reliance on manual inspection, which depends heavily on the availability of domain experts. This reliance significantly slows down the detection process, hindering timely intervention and effective disease management. Moreover, such manual examination of plants is vulnerable to errors. Therefore, there exists a demand to develop an effective and reliable automated approach capable of locating and differentiating among various infections of leaves.

In the next revolution industry, Artificial Intelligence (AI) has the potential to transform agribusiness by allowing producers to achieve more efficient outcomes with minimal effort. Additionally, AI offers a wide range of benefits, including improved resource management, enhanced disease detection, and optimized decision-making processes. One of the cutting-edge innovations in AI, Deep Learning (DL) approaches have outperformed conventional machine learning technologies in several fields due to their robustness to effectively capture the structural information of a sample. Building on these advancements, the emergence of Convolutional Neural Networks (CNNs) has significantly improved the early diagnosis of diseased plant samples Tian et al.^1^. CNNs can learn increasingly complex features as they progress through layers. The incidence of various image distortions i.e., color, light, size, orientation changes, and similarity in the healthy and diseased portions of examined samples remain major challenges in the recognition of various plant leaf infections. Moreover, achieving success in DL requires vast amounts of data to effectively train models and enable them to learn meaningful patterns and knowledge. To address these issues, solutions such as transfer learning or less supervised approach have been proposed. Transfer learning leveraging knowledge from one domain to adapt to a new domain, thereby reducing the need for extensive labeled data. Other approaches such as semi-supervised and self-supervised learning, require only few samples or even raw samples without explicit annotations. For the semi-supervised approach, the primary challenge lies in effectively utilizing the available unlabeled dataset (Li et al.^2^). The authors highlight that selecting pseudo-labeled samples (assigning label to unlabeled data) is a double-edged sword. While a sufficient number of reliable pseudo-labels can compensate for the scarcity of original labeled data, an excessive presence of incorrect labels can negatively impact overall performance. Conversely, being overly cautious and selecting only a few pseudo-labeled samples results in minimal improvement. It is so important to determine the strategy to select certain samples.

The classification of plant leaf disease has recently attracted the attention of the deep learning research community due to its substantial practical applications. However, a challenge with deep learning is that it requires a large amount of data for training, while labeling the data is time-consuming and labor-intensive. Indeed, for these approaches to be effectively applied in real-world scenarios, achieving a low error rate and high precision is crucial. In this study, we propose an Ambiguity-Aware Semi-Supervised Learning for Leaf Disease Classification (AaSSL). Our contributions are summarized as follows:

- We propose a novel Ambiguity Rejection for Semi-supervised learning (ARS) algorithm to mitigate selection ambiguity and enhance the precision of pseudo labels, thereby improving the effectiveness of semi-supervised learning.
- We propose Ambiguity Rejection to further boost the Precision (ARP) of the final classifier by eliminating the ambiguous results. By filtering out these uncertainties, ARP improves the overall precision and reliability of the final classifier.
- We conducted a comprehensive evaluation of the proposed method on two public datasets, coffee and banana, assessing its effectiveness and comparing its performance against state-of-the-art fully supervised learning approaches.

## Related works

Leaf disease classification is a fundamental task in agriculture, alongside other applications such as leaf identification, leaf segmentation, and leaf retrieval. A comparative study of these approaches can be found in Demilie et al.^3^ and Wang et al.^4^. As is common in computer vision tasks, existing approaches for leaf disease classification can be broadly categorized into two main groups: hand-crafted features and deep learning-based methods.

For hand-crafted feature-based methods, one of the first works in this area is by Wang et al.^5^. Initially, images were grouped by clustering color in the Hue Saturation Value (HSV) space. Following this, 50 features-including 21 color features, 4 shape features, and 25 textures-were extracted. A similar idea of using color features was employed in the work of Patil et al.^6^ for the extraction of tomato leaf diseases. Another deep analysis of visual information was conducted by Siricharoen et al.^7^, where nine lightweight sub-features from texture (homogeneity, correlation, entropy), color (mean, standard deviation, skew, visual perception contrast), and shape (hydraulic radius, elongation) were analyzed and extracted as the feature representation. This application achieved 88% accuracy within a few seconds of image acquisition. A more comprehensive analysis of utilizing different visual characteristics of plants is discussed in the work of Pralhad et al.^8^. Another type of information used is the leaf vein pattern, as utilized in Nam et al.^9^ for leaf image retrieval. Instead of using a single-step classification, Tian et al.^10^ classified three features (color, texture, shape) using a multiple classifier system (comprising K multiple classifiers) into different mid-level categories, which partially describe the plant’s symptoms. In the second step, features from each mid-level category were extracted and input into an SVM to train and correct errors, further improving performance. In the study of Nancy et al.^11^, K-means was used to divide cucumber leaves into different groups, followed by the use of Gray Level Co-occurrence Matrix (GLCM) to extract 13 different features. The Random Forest classifier was then used to make decisions based on the extracted features. These approaches have notable drawbacks, including high sensitivity to new samples when capturing conditions change. Additionally, their robustness remains unverified on larger datasets, as most evaluations have been conducted on limited sample sizes. As a result, research has increasingly shifted toward deep learning approaches, which offer more robust feature extraction capabilities.

With advancements in DL, CNNs have been applied in plant visual symptom assessment, both in terms of accuracy and processing time. In Lee et al.^12^, CNNs were employed to classify the MalayaKew dataset, which comprises 44 classes. The authors indicated that the performance of CNNs outperformed hand-crafted features. A comparative study between hand-crafted features and deep CNNs can be found in Hall et al.^13^. In, Shah et al.^14^ utilized dual CNNs to jointly learn both shape and texture characteristics. Specifically, two separate CNN branches were used to process the leaf image (the entire image) and the texture patch (an enlarged and center-cropped version of the original leaf image) to extract distinct features. The final feature representation was obtained by concatenating the outputs of both branches. In Hu et al.^15^, the authors proposed a multi-scale fusion CNNs for the identification of plant leaves. Building on the idea that different image resolutions capture distinct features, input images were downscaled to multiple sizes, with each size processed by separate CNNs to extract features. The final representation was obtained by fusing these multi-scale features, enhancing the model’s ability to capture diverse visual patterns. Instead of using only one network for feature extraction, ensemble techniques have been employed to leverage different models, as seen in Pham et al.^16^, Sarawathi et al.^17^, and Fenu et al.^18^. In Pham et al.^16^, empirical studies with different backbone CNNs using both early and late fusion were conducted on coffee leaf classification. The results indicated that depending on the fusion method, different combinations could achieve similar performance. Instead of randomly choosing two different networks, Fenu et al.^18^ used four convolutional neural networks for training, selecting the best two models as candidates for the ensemble phase. The final prediction was made using a trainable weighted combination of the two models.

While previous approaches relied heavily on available ground truth, there are cases where collecting samples is quite difficult. Therefore, methods need to be developed to handle less-manually labeled data, as discussed in Rezaei et al.^19^, Li et al.^2^, Wu et al.^20^, and Argueso et al.^21^. Few-shot learning (FSL) aims to train models that can generalize from a limited number of training examples. The key concept of this approach is to define how many samples are needed to fine-tune the model. In Argueso et al.^21^, a CNN was first trained to extract features with a shallow Support Vector Machine (SVM) as the classifier from a source domain dataset. In the target domain, the CNN was frozen, and the SVM classifier was fine-tuned. A deep ablation study on the number of fine-tuning samples was conducted, ranging from 1 to 140, achieving 90% accuracy with 80 samples. In Rezaei et al.^19^, only 5 samples per class were used. To make the model more robust, meta-learning combined with a novel feature-level attention module was applied to a ViT model.

A semi-supervised approach is also interesting. In this approach, a few samples are used to train a prototype model, which is then used to predict new, unlabeled samples. The labeled data is combined with the unlabeled data to further train the model. The most challenging aspect of this approach is selecting new samples to add to the continuous training process. In Li et al.^2^, the authors proposed using a confidence interval to select unlabeled samples for pseudo-labeling. An unlabeled sample is selected only when the prediction confidence is greater than 99.5%. The iterative semi-supervised step was repeated three times to further improve performance. In Laine et al.^22^, Bertherlot et al.^23^, and Miyato et al.^24^, the authors explored consistency regularization of data (between labeled and unlabeled data) by adding the corresponding loss to the training model. This approach assumes that data augmentation transformations or model randomness do not change the prediction results. Another approach is to enrich the training data with unlabeled data, as seen in Lee et al.^25^ and Xie et al.^26^. In Lee et al.^25^, the authors proposed the pseudo-labeling process, where they selected the class with the maximum predicted probability and assigned this label to the sample. The unlabeled and labeled samples were then mixed for continuous training. In Xie et al.^26^, they first trained an EfficientNet model as a teacher to generate pseudo-labels for 300 million unlabeled images and then trained a larger EfficientNet as a student to learn from both true labeled and pseudo-labeled images. An important aspect of the training was balancing the number of pseudo-labeled images in each class. In Pham et al.^27^, parallel training between student and teacher models was proposed. The teacher model aimed to create more accurate pseudo-labels, while the student model learned from them. In general, this approach seeks to enrich training data with a large amount of unlabeled data. To the best of our knowledge, there is no prior work on selecting new samples by analyzing the precision of the pseudo-labeling.

**Fig. 1:**
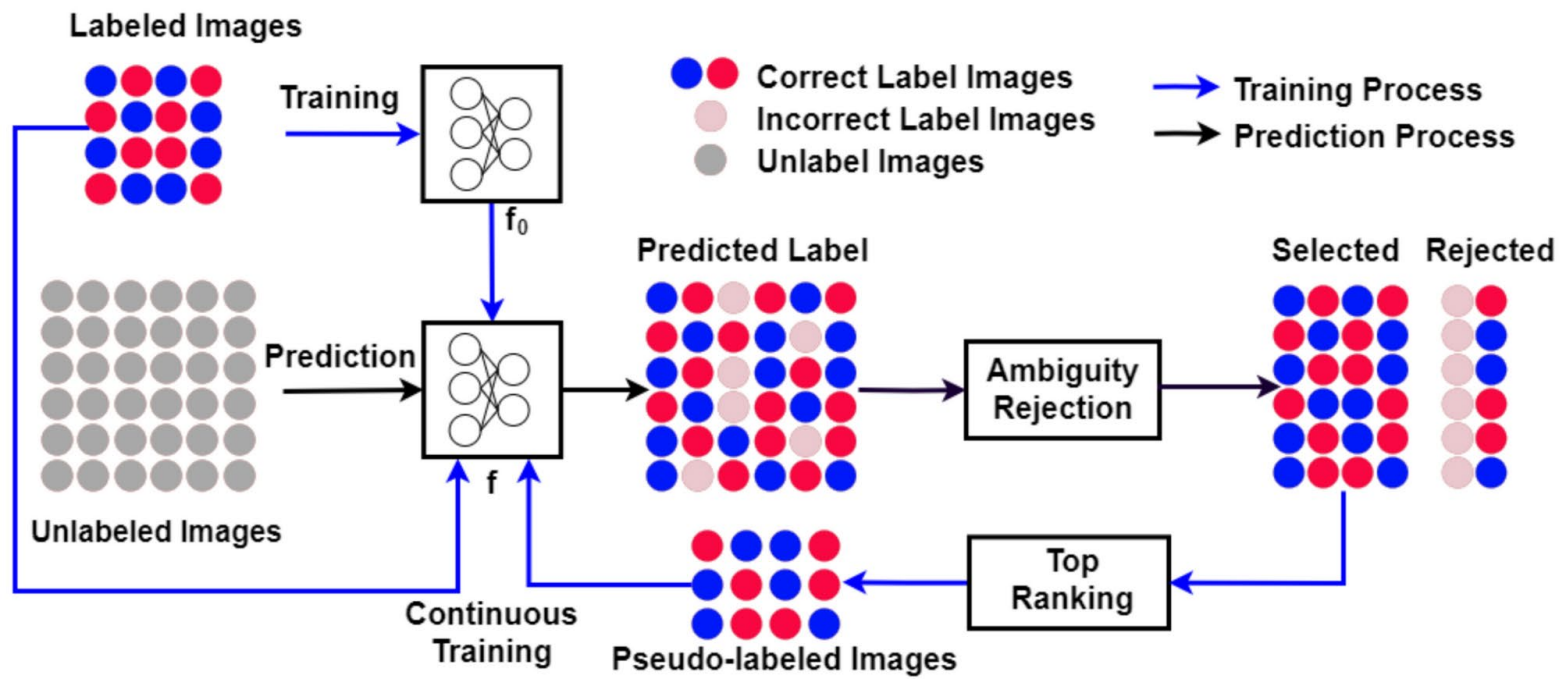
Overview of the proposed ambiguity-aware semi-supervised learning for leaf disease classification. By integrating ambiguity rejection, ambiguous results are eliminated, thereby increasing the precision of both the pseudo-labeled results and the final classification results.

## Method

Our proposed Ambiguity-Aware Semi-Supervised Learning for leaf disease classification (AaSSL) is illustrated in Fig. 1. Our method utilizes a shared per-class ambiguity rejection module to eliminate ambiguous results for the pseudo-labeled samples (ARS) and the final classifier output (ARP). In the initial step, a small set of labeled images is used to train the prototype model, ($f_0$). In the subsequent step, the trained *f* model generate labels for unlabeled images. The ARS module removes noisy results, while the Top Ranking module ensures balanced selection of pseudo-labeled images for the next training cycle. The *f* model is then iteratively re-trained using the refined pseudo-labeled dataset and initial labeled dataset until its performance stabilizes. The trained *f* model from the last iteration serves as the final classifier. ARP is then applied to eliminate ambiguity from the final classifier results.

Our proposed AaSSL uses DeiT^28^, an improved version of the Vision Transformer (ViT)^29^, as the backbone due to the limited number of samples in the training datasets. ViT firstly proposed in the work of Dosovitskiy et al.^29^. By considering the whole image as a sequence of sub-images, this model learns the information from each sub-images then accumulate these information through self-attention to get final representation of image. The challenge with this approach lies in the substantial amount of data required to effectively train large-parameter models like ViT. To overcome this limitation, transfer learning is essential, allowing the model to leverage previously learned knowledge and adapt to a new domain instead of being trained from scratch. DeiT, designed by Touvron et al.^28^, is a data-efficient vision transformer that achieves performance comparable to ViT while requiring significantly less data. Additionally, it is interesting to explore how these large models behave when applied to small datasets. Therefore, we selected DeiT as the backbone for feature extraction. To adapt it to this specific task and different datasets, the original classification head was replaced with a fully connected layer, where the number of outputs corresponds to the number of categories or diseases.

### Semi-supervised learning

Semi-supervised learning is a machine learning paradigm that utilizes both labeled and unlabeled data during training, aiming to improve model performance while reducing the reliance on large labeled datasets. Let $X_l = \{x_1, x_2, \dots , x_l\}$ be the set of labeled data with corresponding labels $Y_l = \{y_1, y_2, \dots , y_l\}$, and $X_u = \{x_{l+1}, x_{l+2}, \dots , x_{l+u}\}$ be the set of unlabeled data points. The goal is to take an advantage of the unlabeled data to improve the model’s performance. This is achieved through a process that involves initially training a model $f_0$ on the labeled dataset $(X_l, Y_l)$, predicting labels $\hat{Y_u}$ for the unlabeled data $X_u$, and then iterative combining these pseudo-labels $\hat{Y_u}$ with the original labeled data $Y_l$ to refine the init model *f*. As mentioned earlier, the key challenge in semi-supervised learning is accurately incorporating new samples from the unlabeled dataset to enrich the training data. This process is crucial for further enhancing the model’s robustness and overall performance. Inspired by the Ambiguity Rejection module of Pham et al.^30^, we propose ARS to enhance the precision of pseudo-labeled images of semi-supervised learning. Additionally, we introduce ARP to mitigate ambiguity results in the final classifier.

### Ambiguity rejection algorithm

Our multi-disease classifier output is typically defined as a set of probabilities $P = \{p_1,p_2,..,p_m\}$ where *P* represents the predicted probabilities of the *m* diseases, and $p_i$ is the predicted probability for the $i^{th}$ disease. The classifier’s final output of a given image *x* is defined as a function $f(x) = argmax(p_i), \text {where } i \in \{1,2,..,m\}$. When we implement a per-disease confidence threshold to eliminate confusion regions, the function *f*(*x*) is adjusted as shown in Equation (1):

1 $$\begin{aligned} f(x)={\left\{ \begin{array}{ll} reject & \text { if } p_i \le \lambda _i\text {,} \forall i \in \{1,2,..,m\}\\ argmax(p_i), i \in \{1,2,..,m\} & \text { otherwise} \end{array}\right. } \end{aligned}$$

where $\lambda = \{\lambda _1,\lambda _2,..,\lambda _n\}$ denotes confidence thresholds, of which $\lambda _i$ is the threshold of $i^{th}$ disease ($d_i$). This algorithm estimates a threshold for each disease and utilizes it to determine whether a new sample should be accepted, ensuring more reliable pseudo-labeling. In this study, we utilize the validation dataset to estimate the threshold $\lambda _i$ for the disease $d_i$. Specifically, we use the classifier to compute a set of probabilities *P* for each image in the validation dataset. For a given disease $d_i$, we identify potential thresholds ($\lambda _{possible}$), which are the unique values from the probability list $p_{i}$. The key question is how to select the best threshold $\lambda _{best}$ for disease $d_i$. For a chosen threshold $\lambda _i \in \lambda _{possible}$, we identify the rejected samples. If we have *n* rejected samples and *k* of them are incorrectly rejected (i.e., samples that were correctly classified by the classifier), the probability of having at most *k* incorrect rejections is denoted as *ProbFailure*(*k*, *n*). A given $\lambda _{i}$ is deemed acceptable when $ProbFailure(k, n) \le 1 - \beta$, where $\beta$ is the specified significance level. Otherwise, if $ProbFailure(k, n) > 1 - \beta$, it means that there are too many incorrect rejections in the rejected region, exceeding the acceptable threshold. This indicates that the rejected region contains too many correctly classified samples, meaning it is not truly suitable for rejection, as it excludes too many correctly classified samples. Therefore, such a rejected region cannot be accepted. For each acceptable $\lambda \in \lambda _{possible}$ , we calculate the select accuracy and coverage, and the threshold $\lambda _{best}$ for disease $d_i$ is the one that maximizes both select accuracy and coverage (highest number of selected images). In this research, *ProbFailure*(*k*, *n*) is estimated using the Binomial Cumulative Distribution Function (B-CDF) as shown in Equation 2, where *p* is the probability that a image is wrongly rejected (any rejected image may randomly be wrong so $p = 0.5$).

2 $$\begin{aligned} ProbFailure(k, n) = \text {binom.cdf}(k;n,p)= \sum _{i=0}^{k} \left( {\begin{array}{c}n\\ i\end{array}}\right) p^i(1-p)^{n-i} \end{aligned}$$

where:

- *n* is the number of trials (the number of rejected samples)
- *k* is the maximum number of incorrectly rejected samples.
- *p* is the probability of a sample being incorrectly rejected.
- $$\left( {\begin{array}{c}n\\ i\end{array}}\right)$$ is the binomial coefficient, calculated as $\frac{n!}{i!(n-i)!}$. It represents the number of ways to choose *i* incorrectly rejected samples out of *n*.

Algorithm 1 optimizes the threshold $\lambda _i$ of the $i{\rm th}$ disease to maximize the selected accuracy. For a given $\beta$, we have $\lambda$ set of thresholds for all diseases when applying the algorithm for all diseases. The higher the $\beta$, the smaller the select accuracy. The parameter $\beta$ is chosen based on the data and requirements of the problem. In this study, both the ARS and ARP modules utilize this ambiguity rejection algorithm.

**Algorithm 1:**
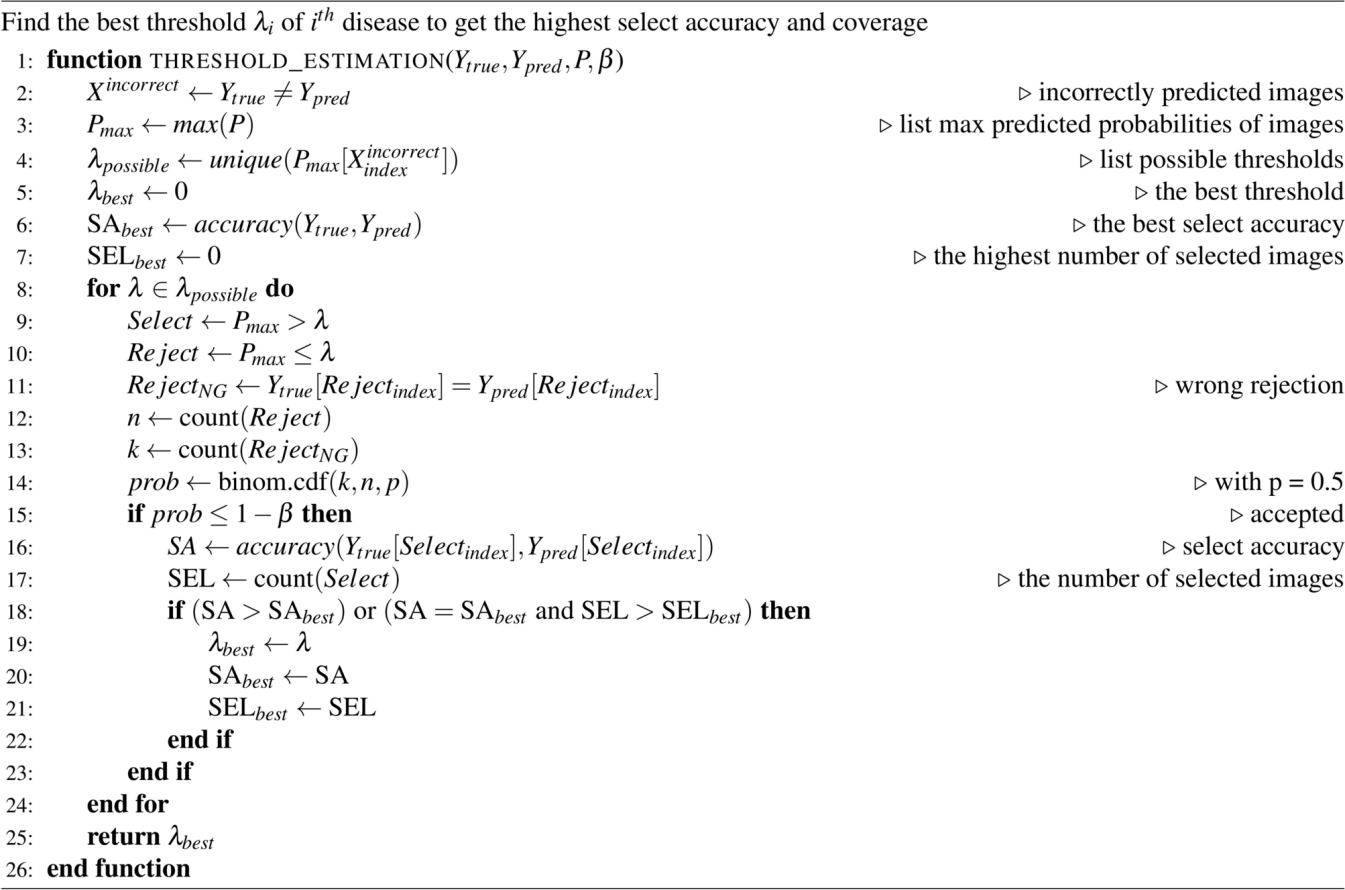
Ambiguity rejection algorithm.

ARS algorithm: is developed from ambiguity rejection algorithm, is followed by the Top Ranking module, which ensures a balanced selection of pseudo-labeled samples for re-training the model in the subsequent steps of semi-supervised learning. As highlighted from Xie et al.^26^, maintaining a balanced distribution of pseudo-labeled images across different diseases is crucial for achieving optimal model performance. Therefore, only top K highest probability images are choose in each step to avoid the redundancy. K needs to be big enough to avoid the stagnation. The top K pseudo-labeled images and labeled images are combined to further fine-tuning model in the iterative manner. The pseudo code of ARS is illustrated in Algorithm 2.

**Algorithm 2:**
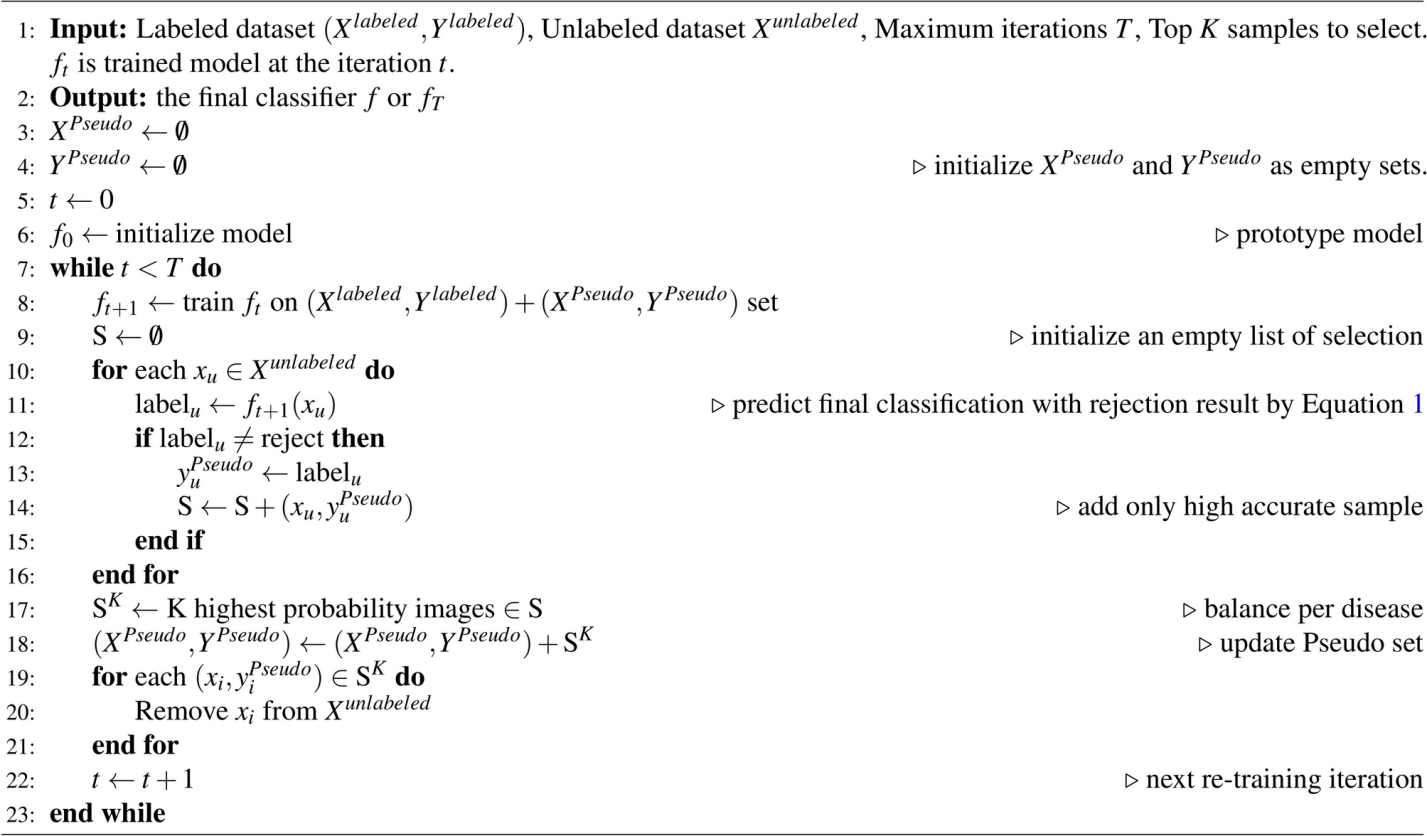
Ambiguity rejection for semi-supervised learning.

ARP algorithm: Practical applications demand highly reliable results with minimal error rates. In leaf disease diagnosis, the results directly influence subsequent treatment decisions; a misdiagnosis could lead to wasted money, time, and failure to cure the disease. Therefore, in this study, we apply ambiguity rejection to eliminate ambiguous results, thereby increasing the precision (select accuracy) of the outcomes and reducing the error rate.

## Dataset

In this study, we used two publicly available datasets about leaf disease classification to evaluate our proposed algorithm, namely BRACOL^31^ and Banana leaf^32^ (available download at https://universe.roboflow.com/ctu-beuuq/identify-diseases-banana-plant).

### BRACOL leaf disease dataset

**Fig. 2:**
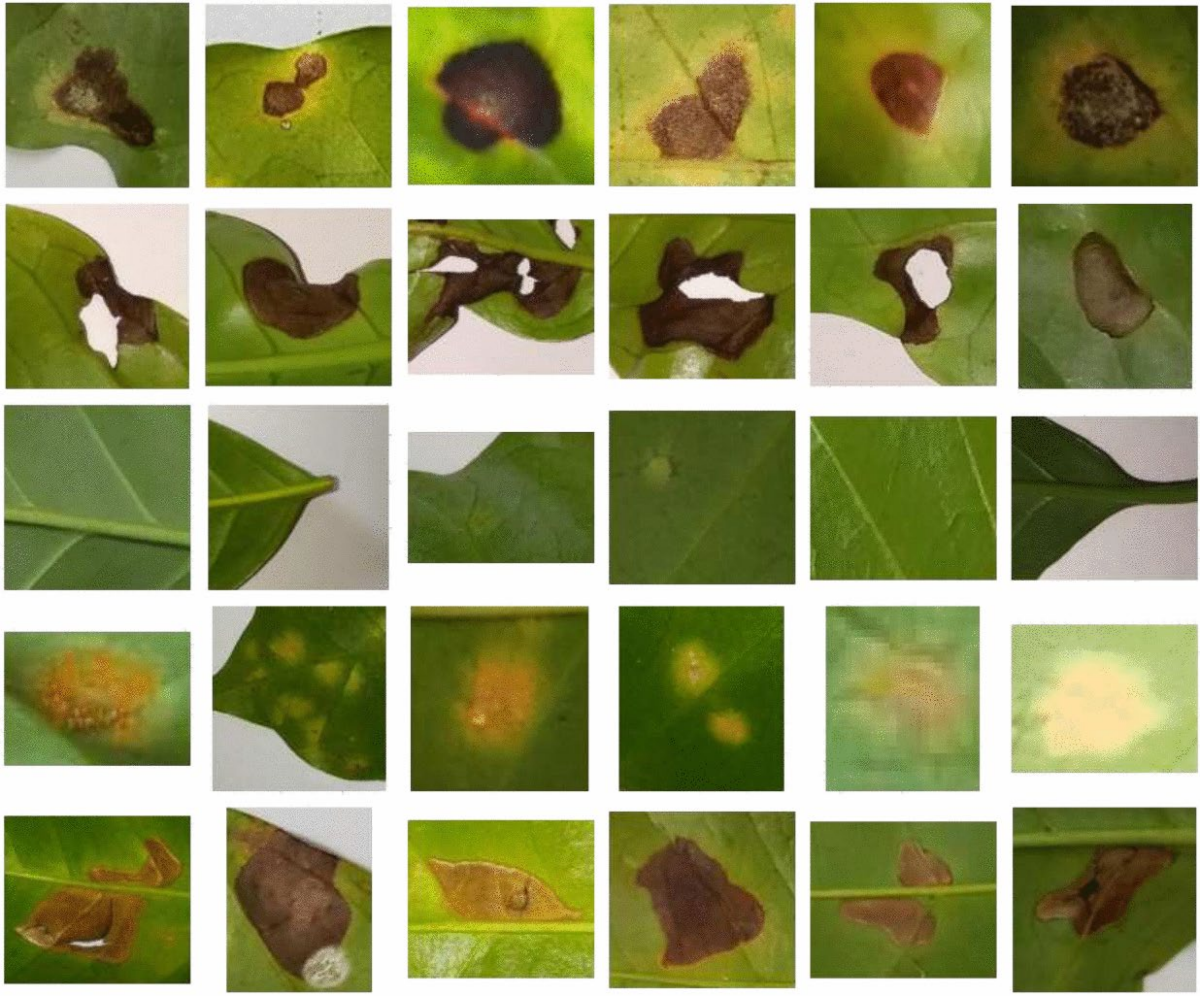
Examples of symptom images of BRACOL dataset.

BRACOL dataset^31^ is a manual-annotated corpus consisting of Leaf and Symptom sets to evaluate Arabica Coffee leaf disease classification and diagnosis approaches. Within the scope of our research, we focus solely on the specify disease classification task in which each sample image contains only one symptom. This dataset whose statistical details of each disease are presented in Table 1. The Symptom set is derived from the original dataset by cropping and isolating specific stress symptoms where each image contains only one stress symptom. Fig. 2 presents some of the symptoms extracted from the original images. We used the same split as in other research^16,31^ for training, validation, and testing and organized into five sub-folders corresponding to the different stress categories: Rust, Miner, Cercospora, Phoma, and Healthy. We keep the same data structure as described below and apply our proposed models to predict the test set and use the same evaluation metrics from the benchmark to make our works more comparable.

**Table 1:** BRACOL leaf disease dataset.

	Diseases	Train	Validation	Test	Total
BRACOL	Rust	694	148	149	991
	Miner	414	89	90	593
	Cercospora	265	56	57	378
	Phoma	353	76	75	504
	Healthy	179	39	38	256
	**Total**	1905	408	409	2722

### Banana leaf disease dataset

**Table 2:** Banana leaf disease dataset.

	Diseases	Train	Validation	Test	Total
Banana	Cordana	237	85	63	385
	Sigatoka	740	227	165	1,132
	Pestalotiopsis	277	77	61	415
	Healthy	144	41	34	219
	**Total**	1398	430	323	2151

Banana leaf disease dataset^32^ is open public dataset. This dataset consists of 2151 cropped disease leaf images belonging into 4 categories (Healthy, Cordana, Pestalotiopsis, Sigatoka). Similar with BRACOL dataset, each image contains only one of the below disease. We used the same train, validation and test set as their provide in the Table 2.

**Fig. 3:**
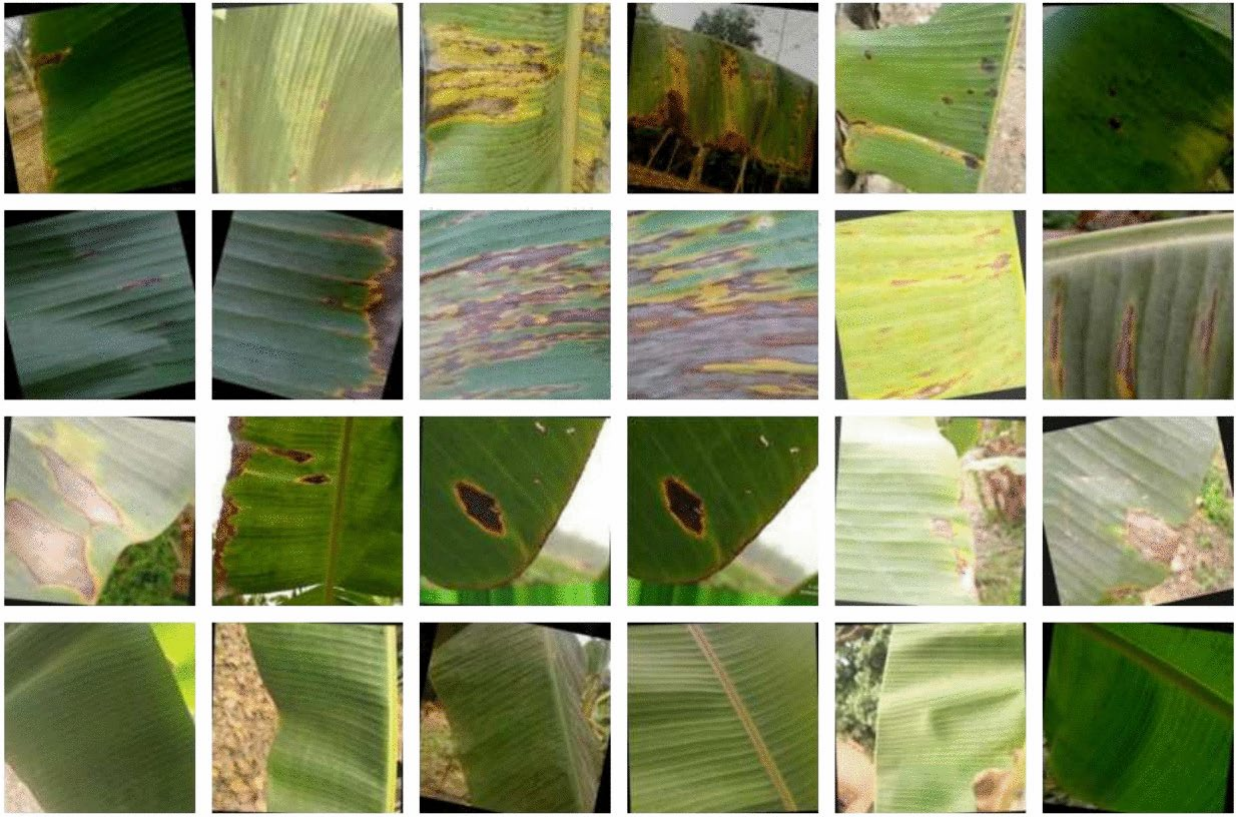
Examples of symptom images of Banana dataset.

A few samples of this dataset can be found in the Fig. 3.

## Experimental results

In this section, we present the evaluation metrics as well as the configuration that we use to evaluate our proposed method. Moreover, several experiments have been conducted to see the tendency with additional analysis.

### Evaluation metrics

Similar to other classification problems, we use Precision, Recall, Accuracy, and F1 Score to evaluate performance of model. Additionally, we also use Coverage (COV) and Select Accuracy (SA) as in Pham et al.^30^. Select accuracy (SA) represents the accuracy on the subset of samples the model is confident about, while Coverage (COV) refers to the proportion of total samples for which the model provides a confident prediction. There is an inherent trade-off between SA and COV: while a high SA with high COV is ideal, an excessively high SA with very low COV indicates that the model is only confident in very few cases, limiting practical usability. In this study, we aim for an SA of approximately 99%, which effectively ensures no errors in the model’s confident predictions. We then evaluate the corresponding COV to determine how many samples the model can confidently classify while maintaining this high level of accuracy. Striking a balance between SA and COV is crucial to ensuring both reliability and broad applicability in real-world scenarios. These metrics are defined as follows:

3 $$\begin{aligned} \text {Precision}= & \frac{TP}{TP + FP} \end{aligned}$$

4 $$\begin{aligned} \text {Recall}= & \frac{TP}{TP + FN} \end{aligned}$$

5 $$\begin{aligned} \text {Accuracy}= & \frac{TP + TN}{TP + TN + FP + FN} \end{aligned}$$

6 $$\begin{aligned} \text {F}_1= & 2 \cdot \frac{\text {Precision} \cdot \text {Recall}}{\text {Precision} + \text {Recall}} \end{aligned}$$

7 $$\begin{aligned} \text {COV}= & \frac{|D_{\text {sel}}|}{|D|} \end{aligned}$$

8 $$\begin{aligned} \text {SA}= & \frac{1}{|D_{\text {sel}}|} \sum _{i \in D_{\text {sel}}} \textbf{1}(y_i = \hat{y}_i) \end{aligned}$$

where *TP*, *TN*, *FP*, and *FN* stand for true positives, true negatives, false positives, and false negatives, respectively. All Precision, Recall, and F1 metrics calculate by first evaluating them individually for each disease and then taking the average across all diseases (macro). *D* is the entire dataset. $D_{\text {sel}}$ is the subset of the dataset that the classifier decides to classify (not rejected). $y_i$ is the true label of the image *i*. $\hat{y}_i$ is the predicted label of the image *i*. $\textbf{1}(y_i = \hat{y}_i)$ is the indicator function that equals 1 if the predicted label is correct and 0 otherwise.

### Experimental configuration

For the DeiT model, we used the pre-trained weights from ImageNet-1k dataset Deng et al.  ^33^. Another important parameter is $\beta$ of Ambiguity Rejection for ARS and ARP. It largely depends on the accuracy of the base model and the target select accuracy. Therefore, we select $\beta$ depending on the dataset and the evaluation scenarios, specifically the initial percentage of labeled images. For the Top Ranking module of ARS, we fix the number of added samples *K* to 200 samples per semi-supervised learning step.

### Performance evaluation

#### Ablation study

In this study, we use cross-validation to evaluate the proposed AaSSL for each evaluation scenario of initial labeled images in semi-supervised learning. For instance, in the case of using 5% training dataset as initial labeled images, the remaining 95% is treated as unlabeled images, and used for continuous semi-supervised learning. Accordingly, we apply 20-fold cross-validation, meaning we conduct twenty different runs of semi-supervised learning and aggregate the results.

#### Amount of labeled data for training

We evaluate multiple scenarios, including 5%, 10%, 20%, 25%, 33%, and 50% of the training set as initial labeled images, corresponding to 20-fold, 10-fold, 5-fold, 4-fold, 3-fold, and 2-fold cross-validation, respectively. The results presented are the averages obtained from the folds, respectively. Detailed results are provided in Table 3. The results obtained using 100% of the labeled data represent the average of five independent runs.

**Table 3:** Performance on based on amount of labeled data for training.

Data ratio	Accuracy		Precision		Recall		$\hbox {F}_1$	
	Supervised	Semi-supervised	Supervised	Semi-supervised	Supervised	Semi-supervised	Supervised	Semi-supervised
BRACOL dataset								
05%	$90.46 \pm 1.74$	$92.18 \pm 1.45$	$89.98 \pm 1.58$	$91.43 \pm 1.53$	$89.94 \pm 2.38$	$91.12 \pm 1.87$	$89.14 \pm 2.00$	$91.16 \pm 1.66$
10%	$92.81 \pm 1.63$	$93.94 \pm 0.94$	$92.54 \pm 1.55$	$93.41 \pm 1.12$	$91.54 \pm 2.42$	$93.26 \pm 0.98$	$91.72 \pm 2.21$	$93.29 \pm 1.00$
20%	$93.84 \pm 1.86$	$95.26 \pm 0.66$	$94.17 \pm 1.54$	$94.75 \pm 0.81$	$92.36 \pm 2.66$	$94.94 \pm 0.72$	$92.91 \pm 2.31$	$94.80 \pm 0.74$
25%	$95.17 \pm 0.88$	$95.98 \pm 0.47$	$95.08 \pm 0.49$	$95.57 \pm 0.54$	$94.41 \pm 1.39$	$95.49 \pm 0.47$	$94.66 \pm 1.00$	$95.51 \pm 0.49$
33%	$95.76 \pm 0.61$	$96.33 \pm 0.49$	$95.58 \pm 0.92$	$95.92 \pm 0.64$	$95.16 \pm 0.70$	$95.92 \pm 0.64$	$95.31 \pm 0.76$	$95.94 \pm 0.57$
50%	$95.72 \pm 0.52$	$96.58 \pm 0.69$	$95.56 \pm 0.87$	$96.17 \pm 0.92$	$95.43 \pm 0.08$	$96.33 \pm 0.51$	$95.45 \pm 0.37$	$96.23 \pm 0.71$
100%	$96.78 \pm 0.68$	–	$96.58 \pm 0.83$	–	$96.66 \pm 0.69$	–	$96.60 \pm 0.74$	–
Banana dataset								
05%	$90.74 \pm 2.27$	$94.09 \pm 2.07$	$90.13 \pm 2.26$	$92.13 \pm 2.55$	$86.84 \pm 3.29$	$93.90 \pm 2.28$	$87.89 \pm 3.08$	$92.85 \pm 2.42$
10%	$94.83 \pm 1.95$	$96.87 \pm 1.31$	$94.31 \pm 1.88$	$95.71 \pm 1.89$	$92.86 \pm 3.33$	$97.04 \pm 1.22$	$93.18 \pm 3.13$	$96.30 \pm 1.55$
20%	$97.83 \pm 0.82$	$98.26 \pm 0.35$	$97.21 \pm 0.88$	$97.47 \pm 0.41$	$97.40 \pm 1.12$	$98.20 \pm 0.17$	$97.25 \pm 1.03$	$97.82 \pm 0.25$
25%	$98.30 \pm 0.40$	$98.76 \pm 0.36$	$98.02 \pm 0.20$	$98.34 \pm 0.59$	$97.89 \pm 0.62$	$98.50 \pm 0.79$	$97.94 \pm 0.44$	$98.40 \pm 0.68$
33%	$98.45 \pm 0.31$	$98.76 \pm 0.31$	$98.05 \pm 0.39$	$98.08 \pm 0.62$	$97.99 \pm 0.99$	$98.55 \pm 0.50$	$98.00 \pm 0.71$	$98.31 \pm 0.57$
50%	$98.92 \pm 0.66$	$99.23 \pm 0.22$	$98.83 \pm 0.52$	$98.99 \pm 0.28$	$98.59\pm 0.86$	$99 \pm 0.28$	$98.70 \pm 0.69$	$98.99 \pm 0.28$
100%	$99.31 \pm 0.26$	–	$99.17 \pm 0.30$	–	$99.11 \pm 0.33$	–	$99.14 \pm 0.31$	–

The first observation reveals that the application of continuous semi-supervised learning generally enhances performance compared to purely supervised learning, particularly when using the same amount of initial training data. For instance, with only 5% labeled data, semi-supervised learning improves accuracy from 90.46% to 92.18% in the BRACOL dataset and from 90.74% to 94.09% in the Banana dataset. Similar trends are observed across other performance metrics, including precision, recall, and ${F_1}$. These improvements can be attributed to the model being exposed to more data through continuous learning, thereby increasing robustness. The second observation shows that increasing the percentage of labeled training data yields slight performance gains. For example, in the BRACOL dataset, the accuracy increases from 95.72% to 96.58% with semi-supervised step (using 50% labeled data). We can also see that at that time, this performance almost the same with the use of 100% labeled data in the fully supervised manner (96.58% and 96.78% on BRACOL dataset, 99.23% and 99.31% on Banana dataset) . More, using semi-supervised learning with 20% labeled data (accuracy of 95.26%) can achieve nearly the same performance as 50% labeled data in purely supervised learning (95.72 %). This suggests that a significant reduction in labeled data is possible while still maintaining competitive performance, helping to minimize the costly and time-consuming process of labeling new data. Moreover, the higher standard deviations observed in the 5% and 10% labeled data experiments, especially in the BRACOL dataset, can be explained by the variability in data distribution due to the limited size of the training set used in these cases. This highlights the challenges associated with small training datasets, which can lead to less stable results.

#### Number of iterations in semi-supervised learning

**Fig. 4:**
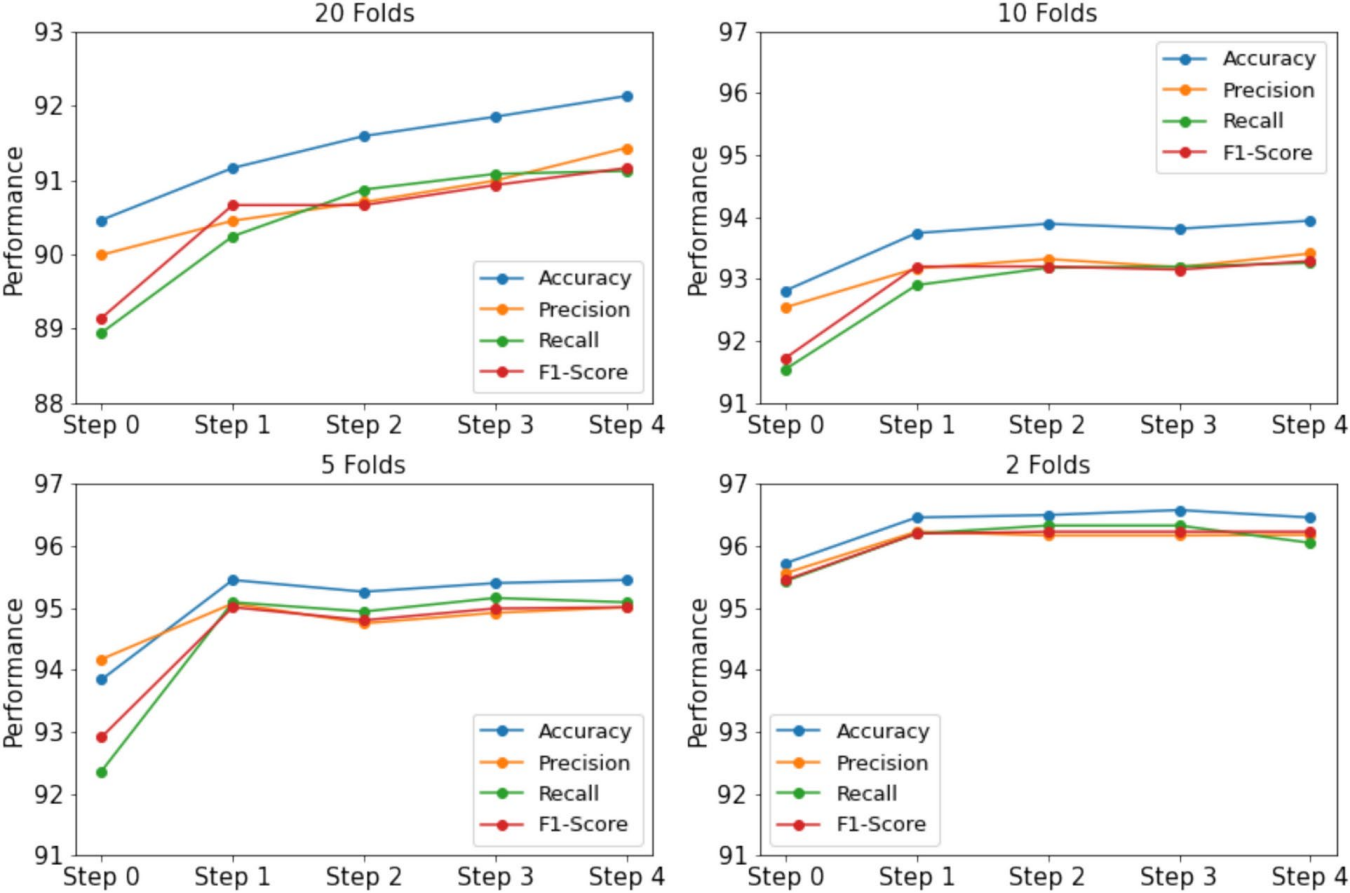
Performance variation across iterations on the BRACOL dataset.

**Fig. 5:**
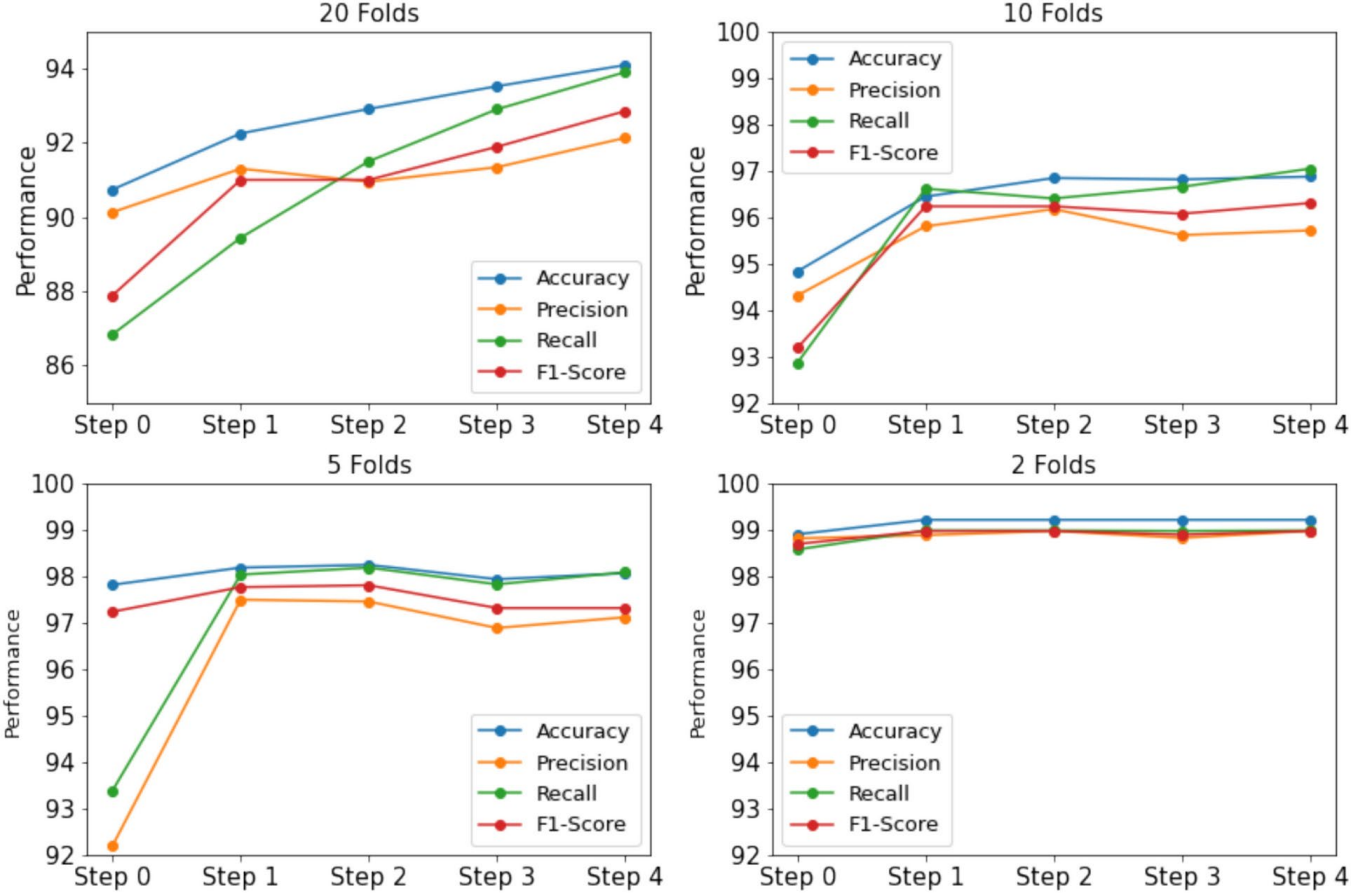
Performance variation across iterations on the Banana dataset.

In this experiment, we aim to evaluate the impact of the number of iterations, denoted as *T*. To analyze this, we examine the performance variations in scenarios where 5%, 10%, 20%, and 50% of the labeled data are used for training. The trends across different configurations are illustrated in the Figs. 4 and 5. When a smaller portion of labeled data is used, such as 5% or 10%, more iterations are required to achieve optimal performance. For example, in case of 5% data of BRACOL dataset, At Step 0, the accuracy starts at 90.46%, and gradually improves with each iteration, reaching 92.13% at Step 4. Similar pattern can found in Banana dataset, accuracy starts at 90.74% in Step 0 and improves significantly, reaching 94.09% at Step 4. Conversely, as the amount of labeled data increases (e.g., 20% or 50%), fewer iterations are necessary to obtain stable results. Furthermore, when the base model already achieves high performance with 50% of the training data, additional iterations yield minimal improvement, with performance remaining close to the baseline. This is because 50% of the labeled data already provides performance close to that of the full dataset, making additional samples have a limited impact on further enhancing the model. Moreover, we also found that in some folds with a small data portion, using more iterations can lead to worse performance compared to the supervised baseline. This occurs because the pseudo-labels of new samples may be incorrect, causing the model to become confused.

#### The effectiveness of the ARS in selecting samples

**Fig. 6:**
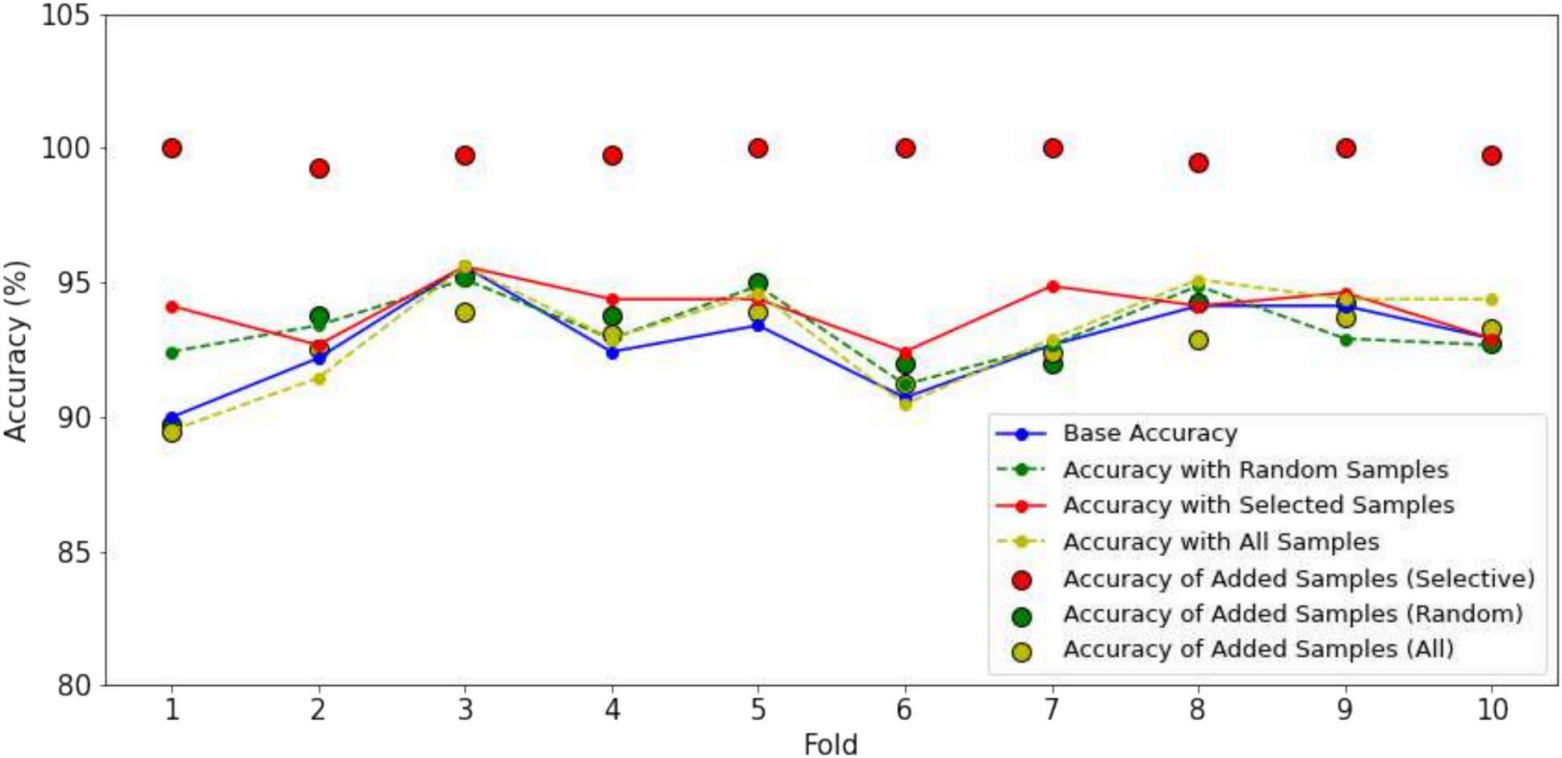
Comparison of accuracy changes with different strategy added samples on the BRACOL dataset.

**Fig. 7:**
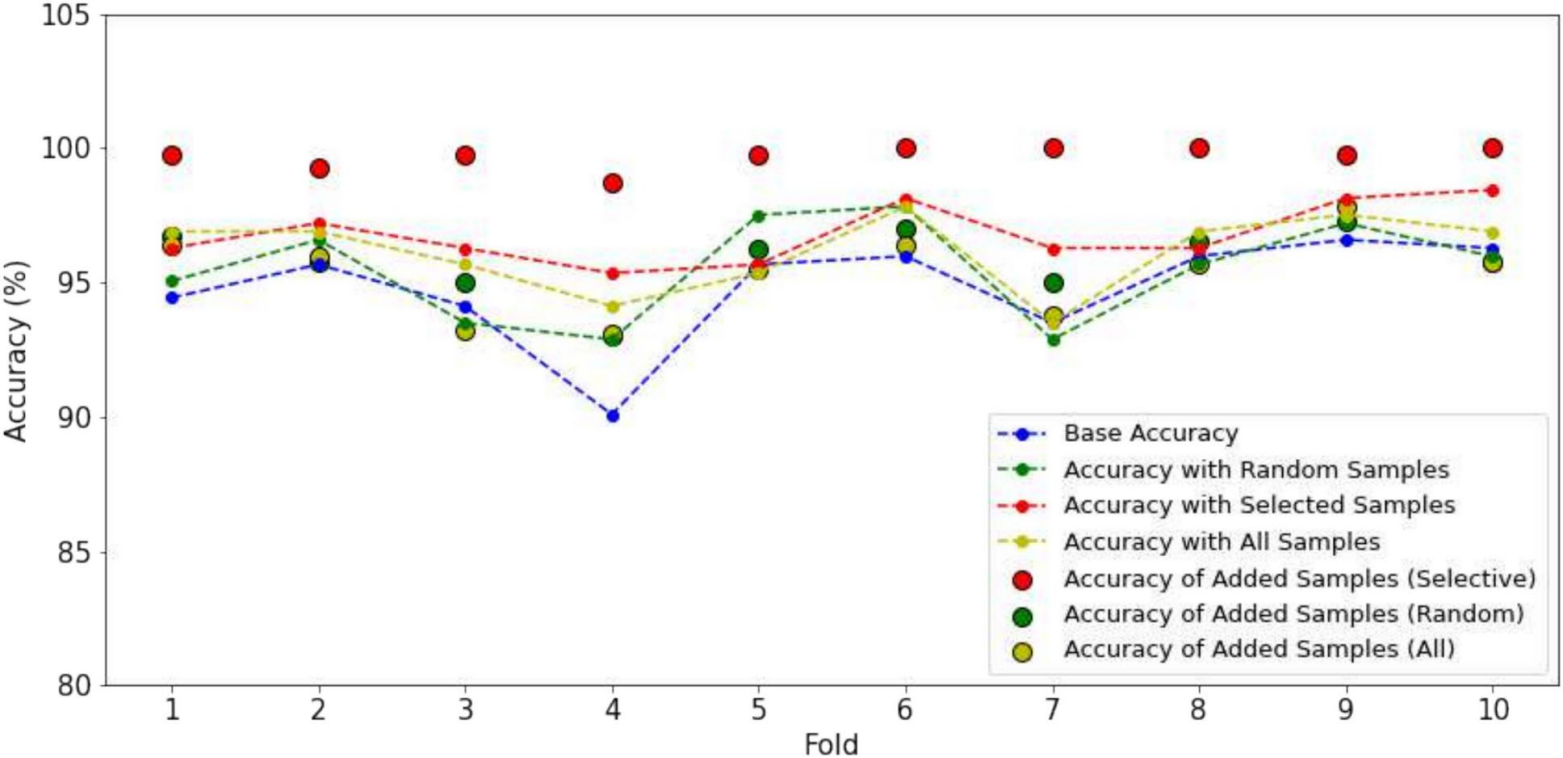
Comparison of accuracy changes with different strategy added samples on the Banana dataset.

In this experiment, we have the comparison between three methods: using a baseline model, adding random samples, and adding selected samples (ARS method) and added all samples. The performance is measured across 10 folds. We fix the baseline to ensure a fair comparison between different sampling methods. The baseline model is trained using 10% of the available data, serving as a consistent reference point for evaluating the impact of adding samples. For two methods (adding random and adding selected samples), we add a fixed number of 400 samples to maintain consistency and evaluate the effectiveness of each method under the same conditions. This setup allows for a clear assessment of how each sampling strategy influences model performance. We test both with BRACOL and Banana dataset. The results can be found in the Figs. 6 and 7.

The baseline accuracy, depicted by the blue line, represents the performance of the baseline model trained on 10% of the data. On the BRACOL dataset, the accuracy of the baseline model varies between approximately 90% and 95% across different folds. The random sampling method, indicated by the green line, demonstrates some improvement over the baseline; however, it lacks consistency. The accuracy of this method fluctuates considerably between folds, occasionally falling below the baseline (e.g., in folds 7 and 9, where random sampling performs worse than the baseline). For the method involving the adding all pseudo-label samples, the results are heavily dependent on the performance of the baseline model. If the baseline model exhibits poor performance, it leads to lower quality in the pseudo-labeling steps, causing confusion within the model. Conversely, when the baseline results are strong, the inclusion of additional samples can enhance performance. Similar trends are observed with the Banana dataset. In contrast, the selected samples method (ARS), represented by the red line, consistently improves accuracy across all folds while maintaining a high level of stability. The accuracy for ARS generally remains between 94% and 96% without significant drops, demonstrating the positive impact of adding high-quality, targeted samples. Additionally, the selected samples method often results in accuracy nearing or reaching 100%, as indicated by the larger red markers representing added sample accuracy. This method proves effective by providing stable and high-performance improvements due to the higher quality and relevance of the added samples. Conversely, both the random sampling method and the addition of all available samples introduce greater variability and sometimes under-perform compared to the baseline, highlighting their lack of reliability. The selected sample method ensures near-perfect accuracy in most cases, whereas random sampling yields less predictable and more fluctuating outcomes.

#### Ambiguity rejection precision

In this experiment, we evaluate the effectiveness of ARP in reducing the error rate of the final classifier by eliminating ambiguous results based on the per-disease thresholds estimated by the proposed ambiguity rejection algorithm. The findings are detailed in Table 4.

**Table 4:** The performance with ambiguity rejection precision.

Data ratio	Base accuracy	Coverage	Select accuracy	Reject accuracy
BRACOL dataset				
05%	$92.18 \pm 1.45$	$72.54 \pm 5.05$	$99.50 \pm 0.48$	$31.58 \pm 4.12$
10%	$93.94 \pm 0.94$	$76.16\pm 2.51$	$99.43 \pm 0.44$	$23.60 \pm 4.06$
20%	$95.26 \pm 0.59$	$82.00 \pm 3.81$	$99.42 \pm 0.41$	$24.11 \pm 4.35$
25%	$95.98 \pm 0.47$	$85.88 \pm 2.00$	$99.08 \pm 0.61$	$22.65 \pm 4.06$
33%	$96.33 \pm 0.49$	$84.03 \pm 4.04$	$99.33\pm 0.14$	$19.57 \pm 1.12$
50%	$96.58 \pm 0.69$	$88.51 \pm 2.76$	$\varvec{99.46 \pm 0.37}$	$25.18 \pm 2.96$
100%	$96.78 \pm 0.68$	$86.16 \pm 4.11$	$\varvec{99.38 \pm 0.24}$	$20.53 \pm 7.01$
Banana dataset				
05%	$94.09 \pm 2.07$	$91.95 \pm 5.23$	$98.56 \pm 0.90$	$61.72 \pm 14.41$
10%	$96.87 \pm 1.31$	$91.12 \pm 2.61$	$99.71 \pm 0.18$	$49.91 \pm 12.10$
20%	$98.26 \pm 0.32$	$96.90 \pm 0.44$	$99.97 \pm 0.16$	$51.64 \pm 6.44$
25%	$98.76 \pm 0.36$	$97.29 \pm 1.46$	$99.76 \pm 0.31$	$49.61 \pm 33.83$
33%	$98.76 \pm 0.31$	$98.04 \pm 2.34$	$99.69\pm 0.31$	$77.78\pm 38.49$
50%	$99.23 \pm 0.22$	$97.99\pm 1.10$	$\varvec{100.0 \pm 0.00}$	$41.67 \pm 11.79$
100%	$99.31 \pm 0.26$	$99.50\pm 0.04$	$\varvec{99.69 \pm 0.02}$	$63.33 \pm 41.50$

Significant values are in bold.

The first observation is the consistent performance of Select Accuracy across both datasets, with values approaching approximately 99%, despite the baseline accuracy only reaching around 92%. This indicates that the model maintains a high level of precision in its predictions, irrespective of the amount of training data used. The stability of Select Accuracy, characterized by its small standard deviation, is evident as it remains relatively unchanged with varying data ratios. Notably, Select Accuracy remains around 99% even with just 5% labeled data for training. In contrast, the Coverage metric, which reflects the model’s ability to handle a larger proportion of the dataset effectively, shows less improvement in the BRACOL dataset, ranging from 72% to 88%. In comparison, the Banana dataset exhibits a more favorable Coverage metric, ranging from 91.95% to 99%. This disparity highlights the robust performance of the model in making confident predictions more effectively. It can be explained by the good performance baseline from Banana dataset in compared with BRACOL dataset.

#### Comparison with state-of-the-art

BRACOL dataset: In the table 5, we present a comparison the performance of the performance of the proposed method against state-of-the-art approaches on the BRACOL dataset.

**Table 5:** Comparison with the state-of-the-art methods on BRACOL dataset.

Method	Accuracy	Precision	Recall	$\hbox {F}_1$
Co-occurrence Matrix (GLCM)^34^	56.50	56.50	56.50	56.50
Local Binary Patterns (LBP)^34^	84.75	84.75	84.75	84.75
Resnet50^35^	97.07	96.85	96.99	–
DeiT^28^	96.78	96.58	96.66	96.60
Early Ensemble^16^	**97.80**	**97.45**	**97.92**	–
Late Ensemble^16^	**97.80**	**97.54**	**97.70**	–
The proposed method (DeiT with 50% label data)	96.58	96.17	96.33	96.23

Significant values are in bold.

The first observation, the results with deep learning based consistently outperform traditional feature-based methods like GLCM^34^ and LBP^34^. The best accuracy achieves by Ensemble methods, around  97% accuracy indicating the effectiveness of ensemble learning for this task. The second observation related to the use of number of labeled data to training model. The results revel that our proposed method with 50% labeled data achieves an accuracy of 96.57% which is comparable with 100% labeled data (96.78%) with the same baseline model. It proves the efficiency of the proposed method in exploring the unlabeled data to keep the high accuracy. The last observation, it is the comparison between CNN-based and Transformer-based. With the same amount data (quite small), the fine-tuning with Resnet50 is more efficient with DeiT. This one can be explained by the small number of parameters of Resnet 50 in comparison with DeiT.

Banana dataset: For this dataset, since there is no research done on it, so here we compare with different backbone CNNs. The results can be found in the Table 6. Similar on BRACOL dataset, all models are trained in fully supervised with all training set (except the proposed model with using only 50%).

**Table 6:** Comparison with the state-of-the-art methods on Banana dataset^32^.

Method	Accuracy	Precision	Recall	$\hbox {F}_1$
Mobilenetv2^36^	99.57	99.50	99.43	99.46
Efficient^37^	99.50	99.30	99.35	99.32
VGG16^38^	99.44	99.31	99.27	99.29
Resnet50^39^	**99.69**	**99.61**	**99.64**	**99.62**
DeiT^28^	99.31	99.17	99.11	99.14
The proposed method (DeiT with 50% label data)	99.23	98.99	99.00	98.99

Significant values are in bold.

For the Banana dataset, all deep learning-based models achieve an accuracy exceeding 99%, demonstrating the effectiveness of this approach. However, it is important to acknowledge that the test set is relatively small, which allows all models to achieve high performance with ease. This implies that while the models exhibit strong results, further validation on larger datasets is necessary to assess their generalization capabilities. Moreover, a similar trend is observed in the BRACOL dataset. The results obtained using 50% labeled data and 100% labeled data on the same backbone (DeiT) remain comparable. Additionally, models with fewer parameters tend to be more efficient, highlighting the trade-off between model complexity and computational efficiency.

### Errors analysis

**Fig. 8:**
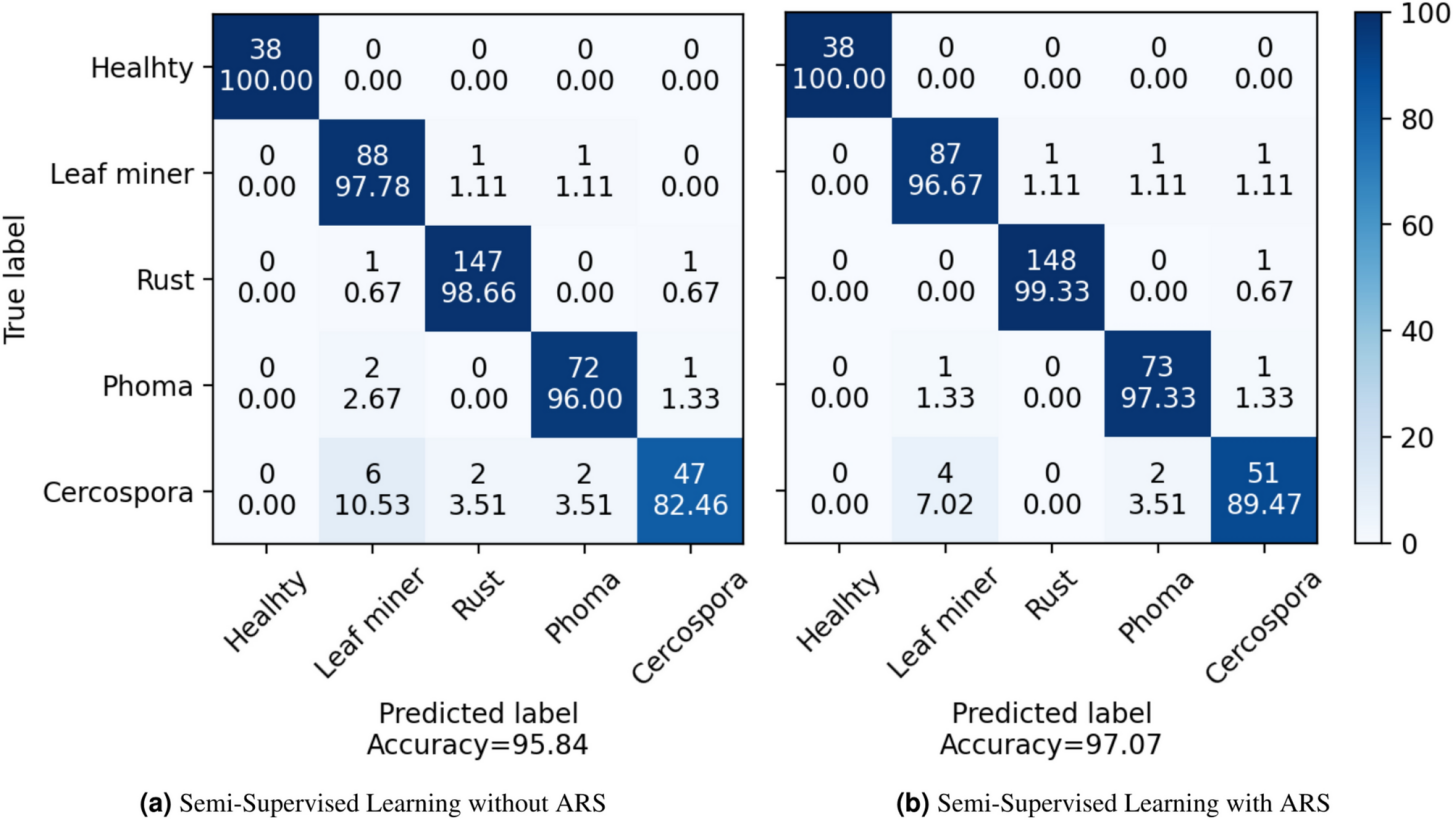
Confusion matrix on BRACOL dataset.

**Fig. 9:**
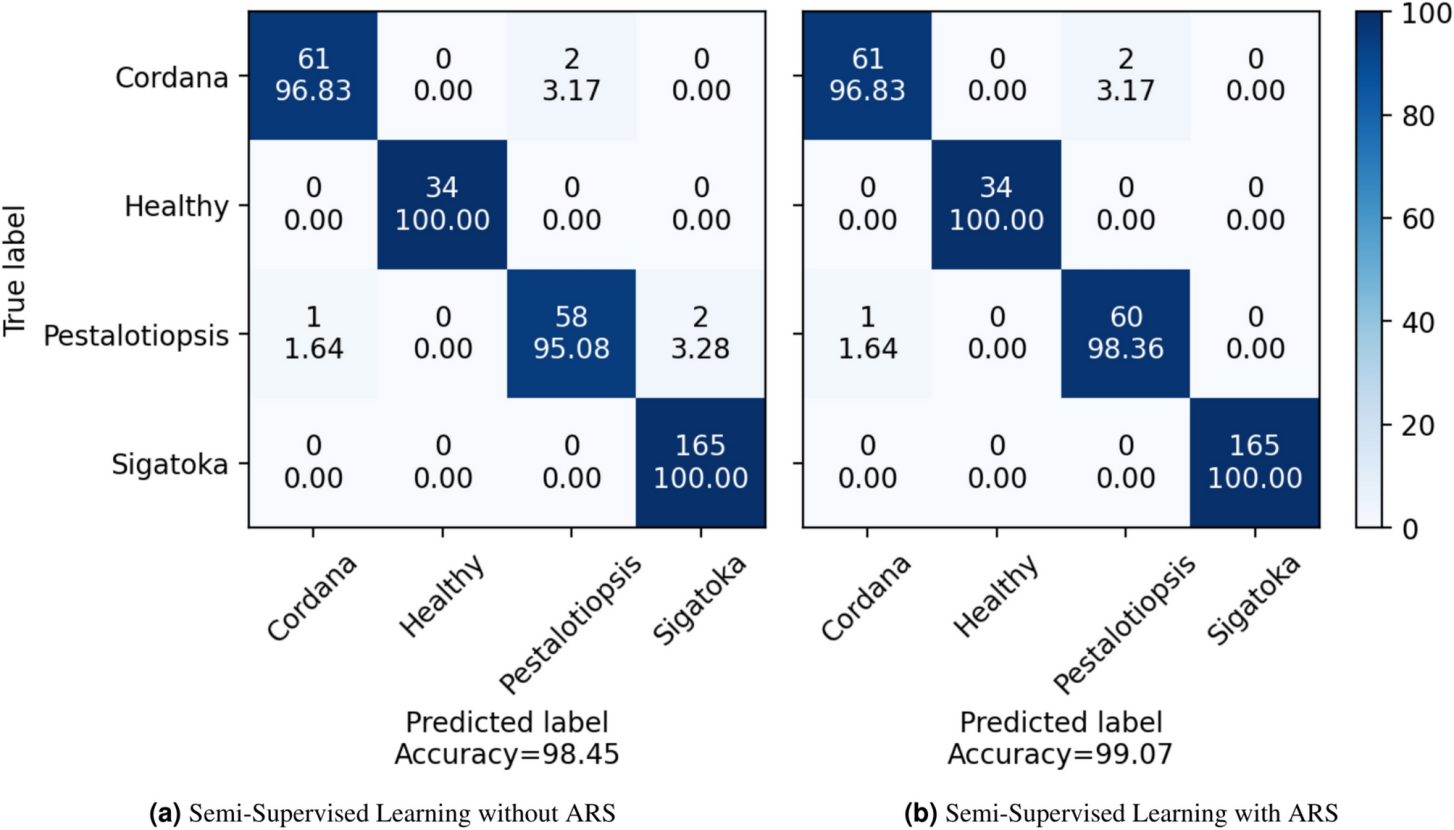
Confusion matrix on Banana leaf dataset.

In this section, we aim to analysis the errors present in both datasets. For the BRACOL dataset, as shown in the confusion matrix in Fig. 8, the ARS training approach effectively reduces errors in the Phoma and Rust categories, with a particularly notable improvement in Cercospora ( 7%) compared to the performance of a purely supervised model. A similar pattern is observed in the Banana Leaf dataset (Fig. 9), where the model demonstrates an additional reduction in errors ( 3% for the Pestalotiopsis category). These results reaffirm the effectiveness of utilizing ARS, as enriching the training data allows the model to significantly minimize errors.

**Fig. 10:**
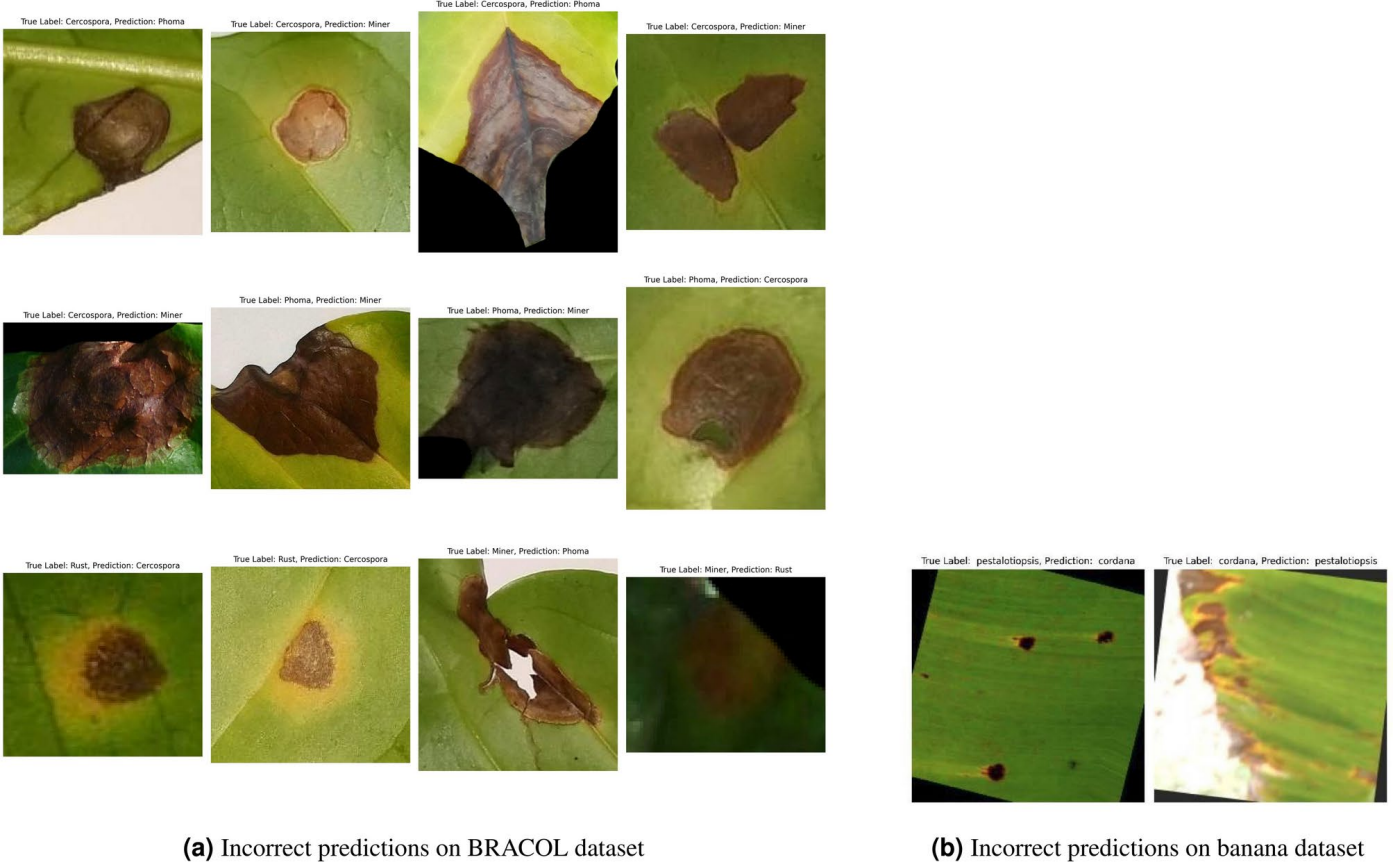
Confusion matrix on Banana leaf dataset.

In the Fig. 10, you can see the visualization in case of the incorrect predictions on BRACOL and Banana dataset. For the BRACOL dataset, most classification errors arise from the confusion between Cercospora and Miner with other classes. Similarly, for the Banana Leaf dataset, misclassifications are primarily limited to just three samples between the Pestalotiopsis and Cordana categories.

## Discussion

As demonstrated in “Experimental results”, the proposed ARS framework can ensure the high accuracy in the process of training model while using less labeled data. These results can largely be credited to ARS’s effective approach in resolving ambiguous outcomes, achieved through a selective pseudo-labeling strategy. By ensuring that only high-confidence pseudo-labels are retained, the iterative training process in the semi-supervised learning framework benefits from a unlabeled set with reduced labeling errors. This, in turn, minimizes noise within the training data and allows the model to focus on more diverse and representative samples, ultimately facilitating better generalization and improved performance. As results in comparison with state-of-the-art methods, it shows only a minor drop ( by using 50% labeled data) compared to fully supervised training. This suggests that the proposed semi-supervised approach is capable of achieving near state-of-the-art performance while significantly reducing labeling requirements. In the case of ARP, by filtering out ambiguous predictions in the final model, ARP achieves a substantial coverage partition exceeding 90%, while maintaining select accuracy at approximately 99%. This further substantiates ARP’s contribution to enhancing the model’s final decision-making process. Despite inherent challenges, these advancements highlight the effectiveness and potential of the proposed approach in achieving superior precision (errors less than 1%) and efficiency in leaf disease classification, especially in scenarios where fully labeled data is limited.

Even though the final selected accuracy can reach 99%, several key issues emerged that affected the overall performance of the proposed method. One major problem was that when the baseline model did not yield strong results, it negatively impacted the subsequent stages of the model. It is challenging to significantly enhance model performance in such cases. Since our method relies on the initial model to accurately identify samples, any deficiencies in the baseline model’s performance could lead to incorrect rejection or acceptance of samples, thus weakening the model’s learning ability. Therefore, careful selection of a appropriate baseline model (based on the amount data) is crucial to ensure the overall effectiveness of the framework. Additionally, the quality of new samples introduced during the learning process played a critical role, as highlighted in the ablation study. If these samples were not representative or inherently ambiguous, they could further degrade the model’s performance. These challenges underscore the importance of having a robust baseline model and ensuring that the new samples are carefully curated to maximize the effectiveness of the ambiguity rejection module.

## Conclusions

In this study, we present a novel AaSSL approach that leverages an ambiguity rejection algorithm to enhance the efficiency of semi-supervised learning in leaf disease classification. By selectively choosing high-quality samples and eliminating ambiguous results, our method significantly enhances the efficiency of semi-supervised learning for leaf disease classification. The proposed ARS method has demonstrated significant utility in selecting reliable samples for further model re-training steps. Remarkably, this strategy achieves competitive results compared to conventional supervised models and, in certain scenarios, even surpasses them when high-accurate pseudo-labeled samples are iteratively incorporated. Notably, by utilizing just 50% of labeled data, we can achieve results comparable to fully labeled data. Additionally, the proposed ARP for final classifier has proven effective in enhancing model precision, achieving a precision above 99% with only 5% labeled data. This demonstrates the potential of AaSSL for real-world applications. It also opens avenues for exploring a broader range of challenges beyond disease classification. Future work will involve evaluating the proposed method on additional datasets to assess its generalizability and effectiveness across various contexts.

## Data Availability

The datasets used and analyzed in our study are available online. If you are unable to download them, please send a request to the corresponding author.

## References

[1] Tian, H., Wang, T., Liu, Y., Qiao, X. & Li, Y. Computer vision technology in agricultural automation-A review. *Inf. Process. Agric.* **7**, 1–19 (2020).

[2] Li, Y. & Chao, X. Semi-supervised few-shot learning approach for plant diseases recognition. *Plant Methods* **17**, 1–10 (2021). 34176505 10.1186/s13007-021-00770-1 PMC8237441

[3] Demilie, W. B. Plant disease detection and classification techniques: A comparative study of the performances. *J. Big Data* **11**, 5 (2024).

[4] Wang, Z., Cui, J. & Zhu, Y. Review of plant leaf recognition. *Artif. Intell. Rev.* **56**, 4217–4253 (2023).

[5] Wang, H., Li, G., Ma, Z. & Li, X. Image recognition of plant diseases based on backpropagation networks. In *2012 5th International Congress on Image and Signal Processing*. 894–900 (IEEE, 2012).

[6] Patil, J. K. & Kumar, R. Color feature extraction of tomato leaf diseases. *Int. J. Eng. Trends Technol.* **2**, 72–74 (2011).

[7] Siricharoen, P., Scotney, B., Morrow, P. & Parr, G. A lightweight mobile system for crop disease diagnosis. In *Image Analysis and Recognition: 13th International Conference, ICIAR 2016, in Memory of Mohamed Kamel, Póvoa de Varzim, Portugal, July 13–15, 2016, Proceedings 13*. 783–791 (Springer, 2016).

[8] Pralhad Mahurkar, D. & Patidar, H. A study of image characteristics and classifiers utilized for identify leaves. In *Intelligent Sustainable Systems: Proceedings of ICISS 2022*. 559–568 Springer, 2022).

[9] Nam, Y., Hwang, E. & Kim, D. A similarity-based leaf image retrieval scheme: Joining shape and venation features. *Comput. Vis. Image Underst.* **110**, 245–259 (2008).

[10] Tian, Y., Zhao, C., Lu, S. & Guo, X. Multiple classifier combination for recognition of wheat leaf diseases. *Intell. Autom. Soft Comput.* **17**, 519–529 (2011).

[11] Nancy, C. & Kiran, S. Cucumber leaf disease detection using glcm features with random forest algorithm. *Int. Res. J. Multidiscip. Technov.* **6**, 40–50 (2024).

[12] Lee, S. H., Chan, C. S., Wilkin, P. & Remagnino, P. Deep-plant: Plant identification with convolutional neural networks. In *2015 IEEE International Conference on Image Processing (ICIP)*. 452–456 (IEEE, 2015).

[13] Hall, D., McCool, C., Dayoub, F., Sunderhauf, N. & Upcroft, B. Evaluation of features for leaf classification in challenging conditions. In *2015 IEEE Winter Conference on Applications of Computer Vision*. 797–804 (IEEE, 2015).

[14] Shah, M. P., Singha, S. & Awate, S. P. Leaf classification using marginalized shape context and shape+ texture dual-path deep convolutional neural network. In *2017 IEEE International Conference on Image Processing (ICIP)*. 860–864 (IEEE, 2017).

[15] Hu, J., Chen, Z., Yang, M., Zhang, R. & Cui, Y. A multiscale fusion convolutional neural network for plant leaf recognition. *IEEE Signal Process. Lett.* **25**, 853–857 (2018).

[16] Pham, T. C., Le, C. H., Packianather, M., Hoang, V.-D. et al. Artificial intelligence-based solutions for coffee leaf disease classification. In *IOP Conference Series: Earth and Environmental Science*. Vol. 1278. 012004 (IOP Publishing, 2023).

[17] Saraswathi, E. & FarithaBanu, J. A novel ensemble classification model for plant disease detection based on leaf images. In *2023 International Conference on Artificial Intelligence and Knowledge Discovery in Concurrent Engineering (ICECONF)*. 1–7 (IEEE, 2023).

[18] Fenu, G. & Malloci, F. M. Classification of pear leaf diseases based on ensemble convolutional neural networks. *AgriEngineering* **5**, 141–152 (2023).

[19] Rezaei, M., Diepeveen, D., Laga, H., Jones, M. G. & Sohel, F. Plant disease recognition in a low data scenario using few-shot learning. *Comput. Electron. Agric.* **219**, 108812 (2024).

[20] Wu, W. Enhanced few-shot learning for plant leaf diseases recognition. *J. Comput. Electron. Inf. Manag.* **11**, 26–28 (2023).

[21] Argüeso, D. et al. Few-shot learning approach for plant disease classification using images taken in the field. *Comput. Electron. Agric.* **175**, 105542 (2020).

[22] Laine, S. & Aila, T. Temporal ensembling for semi-supervised learning. *arXiv preprint* arXiv:1610.02242 (2016).

[23] Berthelot, D. et al. Mixmatch: A holistic approach to semi-supervised learning. *Adv. Neural Inf. Process. Syst.* **32** (2019).

[24] Miyato, T., Maeda, S.-I., Koyama, M. & Ishii, S. Virtual adversarial training: A regularization method for supervised and semi-supervised learning. *IEEE Trans. Pattern Anal. Mach. Intell.* **41**, 1979–1993 (2018). 30040630 10.1109/TPAMI.2018.2858821

[25] Lee, D.-H. et al. Pseudo-label: The simple and efficient semi-supervised learning method for deep neural networks. In *Workshop on Challenges in Representation Learning, ICML*. Vol. 2. 896 (2013).

[26] Xie, Q., Luong, M.-T., Hovy, E. & Le, Q. V. Self-training with noisy student improves ImageNet classification. In *Proceedings of the IEEE/CVF Conference on Computer Vision and Pattern Recognition*. 10687–10698 (2020).

[27] Pham, H., Dai, Z., Xie, Q. & Le, Q. V. Meta pseudo labels. In *Proceedings of the IEEE/CVF Conference on Computer Vision and Pattern Recognition*. 11557–11568 (2021).

[28] Touvron, H. et al. Training data-efficient image transformers & distillation through attention. In *International Conference on Machine Learning*. 10347–10357 (PMLR, 2021).

[29] Dosovitskiy, A. et al. An image is worth 16x16 words: Transformers for image recognition at scale. *arXiv preprint* arXiv:2010.11929 (2020).

[30] Pham, T.-C. et al. Incremental learning and ambiguity rejection for document classification. In *International Conference on Document Analysis and Recognition*. 18–35 (Springer, 2023).

[31] Krohling, R. A., Esgario, J. & Ventura, J. A. BRACOL-A Brazilian arabica coffee leaf images dataset to identification and quantification of coffee diseases and pests. *Mendeley Data* **1** (2019).

[32] CTU. identify-diseases-banana-plant dataset. https://universe.roboflow.com/ctu-beuuq/identify-diseases-banana-plant (2024). Accessed 08 Aug 2024.

[33] Deng, J. et al. Imagenet: A large-scale hierarchical image database. In *2009 IEEE Conference on Computer Vision and Pattern Recognition*.248–255 (IEEE, 2009).

[34] Lisboa, E., Lima, G. & Queiroz, F. Coffee leaf diseases identification and severity classification using deep learning. In *Anais Estendidos do XXXIV Conference on Graphics, Patterns and Images*.201–205 (SBC, 2021).

[35] Esgario, J. G., Krohling, R. A. & Ventura, J. A. Deep learning for classification and severity estimation of coffee leaf biotic stress. *Comput. Electron. Agric.* **169**, 105162 (2020).

[36] Howard, A. G. et al. Mobilenets: Efficient convolutional neural networks for mobile vision applications. *arXiv preprint* arXiv:1704.04861 (2017).

[37] Tan, M. & Le, Q. Efficientnet: Rethinking model scaling for convolutional neural networks. In *International Conference on Machine Learning*.6105–6114 (PMLR, 2019).

[38] Simonyan, K. & Zisserman, A. Very deep convolutional networks for large-scale image recognition. *arXiv preprint* arXiv:1409.1556 (2014).

[39] He, K., Zhang, X., Ren, S. & Sun, J. Deep residual learning for image recognition. In *Proceedings of the IEEE Conference on Computer Vision and Pattern Recognition*.770–778 (2016).

